# Fetal Adrenal Gland in the Second Half of Gestation: Morphometrical Assessment with 3.0T Post-Mortem MRI

**DOI:** 10.1371/journal.pone.0075511

**Published:** 2013-10-07

**Authors:** Zhonghe Zhang, Haiwei Meng, Zhongyu Hou, Jun Ma, Lei Feng, Xiangtao Lin, Yuchun Tang, Xiaoli Zhang, Qingwei Liu, Shuwei Liu

**Affiliations:** 1 Department of Medical Imaging, Shandong Provincial Hospital Affiliated to Shandong University, Jinan, Shandong, China; 2 Research Center for Sectional and Imaging Anatomy, Shandong University School of Medicine, Jinan, Shandong, China; 3 Department of Anatomy, Guilin Medical College, Guilin, Guangxi, China; 4 Department of Histology and Embryology, Shandong University School of Medicine, Jinan, Shandong, China; National Health Research Institutes, Taiwan

## Abstract

**Background:**

The morphometry of fetal adrenal gland is rarely described with MRI of high magnetic field. The purpose of this study is to assess the normal fetal adrenal gland length (AL), width (AW), height (AH), surface area (AS) and volume (AV) in the second half of gestation with 3.0T post-mortem MRI.

**Methods and Findings:**

Fifty-two fetal specimens of 23–40 weeks gestational age (GA) were scanned by 3.0T MRI. Morphological changes and quantitative measurements of the fetal adrenal gland were analyzed. Asymmetry and sexual dimorphism were also obtained. The shape of the fetal adrenal gland did not change substantially from 23 to 40 weeks GA. The bilateral adrenal glands appeared as a ‘Y’, pyramidal or half-moon shape after reconstruction. There was a highly linear correlation between AL, AW, AH, AS, AV and GA. AW, AH, AS and AV were larger for the left adrenal gland than the right. No sexual dimorphism was found.

**Conclusions:**

Our data delineated the normal fetal adrenal gland during the second half of gestation, and can serve as a useful precise reference for anatomy or in vivo fetus.

## Introduction

In contrast to the adult, the fetal adrenal gland is relatively a large organ, which is important to fetal growth [1], [2]. Many diseases may lead to morphological changes in the adrenal gland, such as the fetal adrenal tumors and cystic adrenal masses [1], [3], [4]. As a consequence, it is important to obtain precise quantitative measurements of the adrenal gland during fetal development [1], [5], [6].

At present, ultrasound (US) is the first choice for screening in the clinical examination of fetus [7], but there are many problems associated with the use of two-dimensional (2D) US in assessing the fetal adrenal gland. The method cannot be used to measure the thickness and width of the adrenal gland precisely, nor can it assess the volume of the adrenal gland at different GA. Although three-dimensional (3D) US has been performed, and can provide more accurate results, it is still limited by the common shortcomings of US. In addition, there are still problems with the validation of the measurements, and in particular with reproducibility and accuracy [6].

Prenatal MRI is playing an increasingly important role in the evaluation of fetal development [7]. But it is not always easy to identify abnormalities especially in young fetuses (from 18 to 25 weeks) because at that age the fetus is comparatively small [8]. However, during the later stages of fetal development, the adrenal gland cannot be observed easily because of changes in fetal position and coverage with the maternal organs and fetal limbs [1]. As a consequence, the fetal adrenal gland is not conspicuous or recognizable during the early and late phases of gestation.

The major advantage of extra-uterine MRI is its excellent slice resolution, because it is not restricted in scanning field and sequences [9]. It can clearly describe the normal trajectory and obtain exact quantitative measurements of the fetal adrenal gland. At present, few investigators have quantitatively analyzed the fetal adrenal gland with high field post-mortem MRI, and we still lack data about the quantitative measurements, asymmetry and sexual dimorphism of the fetal adrenal gland.

## Materials and Methods

### Selection of the Specimens

This study was conducted on the approval of Ethical Committee at the School of Medicine, Shandong University (Permit Number: 2012033). The parents’ written consent to donating the fetal cadaver was obtained.

Seventy-two fetal specimens of 23–40 weeks GA were collected in hospitals of Shandong Province. They were derived from medically indicated abortions, spontaneous abortions, fetal deaths or stillbirths, and premature deaths. Because the crown-rump length was more accurate to the GA of the fetus, we first measured the crown-rump length of the fetuses by MRI to provide a more accurate GA, and then measured the fetal adrenal gland. The inclusion criteria were the same as that in our previous research [10]–[14], namely: the maternal pregnancy records did not contain a documented fetal chromosomal abnormality, stressful intrauterine conditions, maternal genetic disease in the family, or a history of seizures in the case of eclampsia; the results of US examination of the fetus during pregnancy and the results of post-mortem MRI examinations of the specimen indicated an anatomically normal and developmentally appropriate fetus.

After application of the selection criteria, 52 specimens remained in the study (the distribution was listed in Table 1).

**Table 1:** GA (weeks) dispositions and numbers of the chosen specimens (n = 52).

GA	Male	Female	GA	Male	Female
23	2	1	32	2	0
24	3	3	33	2	1
25	1	4	34	0	3
26	4	1	35	0	1
27	3	3	36	1	0
28	3	0	37	1	1
29	3	2	38	0	1
30	0	3	39	0	0
31	0	1	40	2	0

### Scanned by 3.0T MR

The specimens were scanned with a SIEMENNS 3.0T MR scanner with a T_2_ weighted 3D SPGR sequence. The wrist coil was used for all the fetuses. Parameters were as follows: Scanning thickness: 0.5 mm, slice interval: 0.5 mm. Repetition time: 1440.0 ms, echo time: 132.6 ms, matrix: 512*512, number of excitations: 4. The scanning time was approximately 30 minutes. The field of view was adjusted according to the circumference of the abdomen to produce a signal to noise ratio of more than 56%.

After scanning, four specimens of 24, 26, 32 and 36 weeks GA were dissected to observe the gross anatomy and make the Nissl-stained sections.

### The 3D Reconstruction

**Figure 1:**
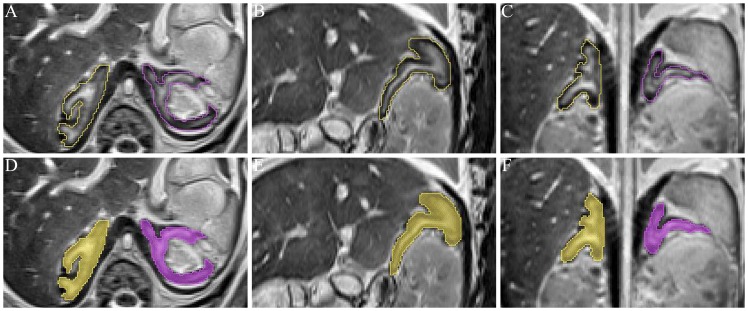
Manual segmentation of the fetal adrenal gland of 36 weeks GA on the T_2_ -weighted MRIs. A, B and C are the transverse, sagittal, and coronal MRIs. Different colors are filled after segmentation (D–F). The fetal adrenal gland is comparatively large, and the inner part is described with high signal intensities and the outer part is low.

**Figure 2:**
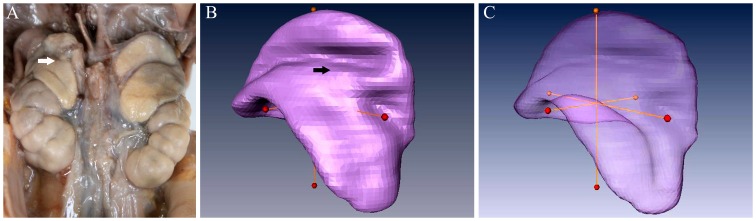
The gross anatomy (A), 3D visualization model and linear measurements of the fetal adrenal gland of 32 weeks GA (B and C). The AL, AW and AH are the longest length of the three axes of fetal adrenal gland (B, C). The fetal adrenal gland is lying and circling upper pole of the kidney, and is relatively a large organ (A). There are good consistencies in delineating the fetal adrenal gland between gross anatomy and the reconstructed 3D model (arrowheads in A and B).

Amira 4.1 software was used for image segmentation and reconstruction. To obtain accurate measurements of the adrenal gland, manual segmentation was carried out on one section (such as the coronal images) and then adjusted on the other two sections (such as transverse and sagittal images). Then three reconstruction models, based on the transverse, coronal and sagittal images respectively, will be obtained for each GA. All the results for fetal adrenal gland in our study will be measured three times. The average will be obtained for each value after ANOVA test. To check the reproducibility of the manual segmentation, the adrenal gland was segmented manually twice simultaneously by two anatomists to obtain a mean value. The time interval between each round of manual segmentation was at least 1 week (Fig. 1). The procedures were as follows. First, the images were imported into Amira 4.1 to be aligned. Second, the fetal adrenal glands were traced around manually on each image in the coronal, sagittal, and transverse planes throughout the series until they could no longer be visualized (Fig. 1). The software then automatically rebuilt the 3D visualization model of the fetal adrenal gland (Fig. 2: B, C).

### Measurements

After the model had been obtained, the AL, AW, and AH were measured. They were recorded as the longest measurements of the three axes of the fetal adrenal gland, respectively (Fig. 2). Finally, the AS and AV of the gland were obtained automatically.

### Statistics

A paired *t* test was used to detect asymmetries in the fetal adrenal glands. The GLM (general linear model) procedure, with GA as the confounding variable, was used to analyze the effect of gender on different measurements of the fetal adrenal gland. Differences were considered statistically significant when the probability *p* was less than 5% (*p*<0.05). The relationship between each measurement and GA was obtained by regression analysis. All statistical analyses were performed using SPSS 17.0.

## Results

### The Fetal Adrenal Gland on Sectional Anatomy and MRI

**Figure 3:**
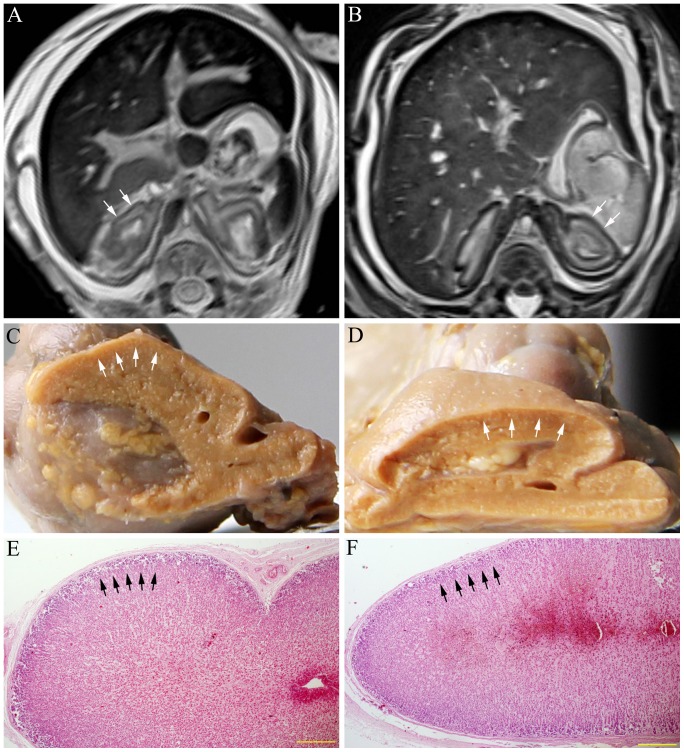
The transverse MRIs, sectional anatomy and Nissl-stained slices of the fetal adrenal gland of different GA. A and B are the transverse MRIs of the fetal adrenal gland of 24 and 36 weeks GA. C and E are the sectional anatomy and the Nissl-stained section of the right adrenal gland in A. D and F are the sectional anatomy and the Nissl-stained section of the left adrenal gland in B. The zona fasciculata, zona reticularis and the medulla of the fetal adrenal gland are described with high signal intensities, and the zona glomerulosa of the cortex is low signal intensity on T_2_ weighted MRI. There are good consistencies in delineating the fetal adrenal gland between the MRI and the transverse sections. The zona glomerulosa of the cortex, containing more cells, becomes thicker as GA increases (arrowheads in A–F). The bars in E, F represent 500 µm.

The adrenal gland is better depicted on T_2_-weighted MRI. The zona fasciculata, zona reticularis, and the medulla of the fetal adrenal gland are described with high signal intensities, and the zona glomerulosa of the cortex is low signal intensity (Fig. 1, 3). The zona glomerulosa of the cortex, containing closely packed cells, becomes thicker as GA increases, which can be observed on the transverse sections and the Nissl-stained slices of the fetal adrenal gland (Fig. 3:C–F). There are good consistencies in delineating the cortex and medulla among the MRI, the transverse sections and the Nissl-stained slices (Fig. 3).

The shape of the right adrenal gland was commonly described as an irregular triangle or pyramid, with a few appearing as a half-moon. Most of the left adrenal glands had a half-moon shape, and a few were pyramidal. They were lying and circling upper pole of the kidney (Fig. 2: A).

**Figure 4:**
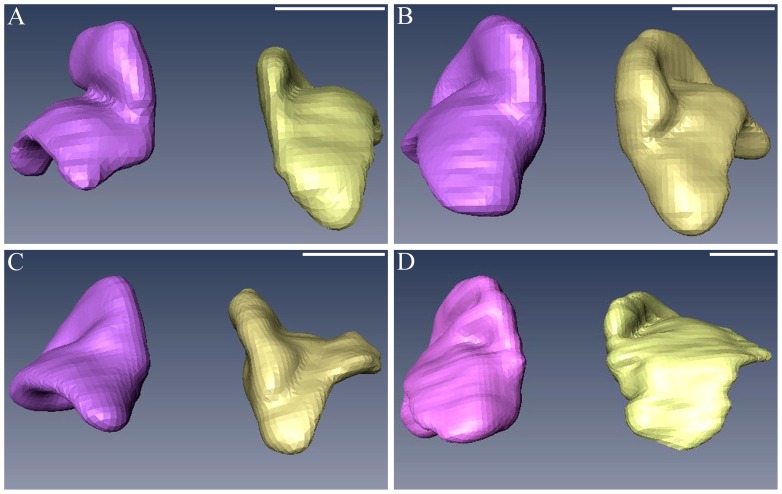
The 3D visualization models of the fetal adrenal gland of different GA. They are 23 (A), 27(B), 32(C), and 36 (D) weeks GA, respectively. The fetal adrenal gland appears as a pyramidal or half-moon shape, and almost remains the same during 23–40 weeks GA. The medial surface is flat. The groove on the lateral surface becomes shallower as GA increases. The bar in each figure represents 1 centimeter.

After 3D reconstruction, shape of the adrenal gland was revealed successfully (Fig. 2), which did not change substantially from 23 to 40 weeks GA (Fig. 4). There are good consistencies in delineating the fetal adrenal gland between gross anatomy and the reconstructed 3D model (Fig. 2: A, B). Compared with the kidney, the fetal adrenal gland is relatively a large organ. (Fig. 2: A). The medial surface was flat and smooth. The groove on the lateral surface, which divided the superior and inferior lobes of the adrenal gland, gradually became shallower as GA increased (Fig. 4), and it can be clearly delineated on the 3D reconstruction model (Fig. 2: A, B).

‘The 3D visualization model of the fetal adrenal gland of 32 weeks GA. Avi’, which can be dynamically displayed, is in the supplement (Movie S1).

### Measurements of the Adrenal Gland

The preliminary intra-observer variability was 0.91. One-way ANOVA with repeated measures indicated that there were no significant differences in the values of AL (*p* = 0.42), AW (*p* = 0.47), AH (*p* = 0.45), AS (*p* = 0.50) and AV (*p* = 0.52) among the coronal, transverse, and sagittal images, which suggested that these measurements could be averaged. Correlations among the coronal, transverse, and sagittal measurements were all high, with all estimates greater than 0.65.

**Figure 5:**
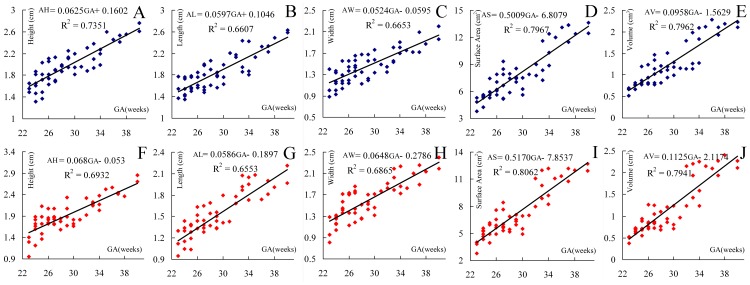
The statistical results between all the measurements and GA. They are the scattergrams, best-fit equations and correlation coefficients (R^2^) of AH (A, F), AL (B, G), AW (C, H), AS (D, I), AV (E, J) and GA. All the measurements linearly increase with GA. Each symbol represents one single fetus. The blue points represent measurements of the left fetal adrenal gland and the red points represent the right.

The relationships between AL, AW, AH (in centimetres), AS (in square centimetres), AV (in cubic centimetres) and GA (in weeks) were all well described by straight lines that linearly increased (Fig. 5). The paired *t* test demonstrated that the asymmetries in the measurements of AL were not significant (*p*>0.05). However, the values for AW, AH, and AS were larger for the left adrenal gland than for the right one (*p*<0.01), so as AV (0.01<*p*<0.05).

The original measurements of the left and right adrenal glands were listed in Table 2.

**Table 2:** The original measurements of the left/right adrenal glands (n = 52).

GA	AH	AL	AW	AS	AV	GA	AH	AL	AW	AS	AV
23	1.49/0.96	1.37/1.30	1.40/0.81	3.81/2.77	0.52/0.38	28	1.87/1.58	1.90/1.69	1.68/1.25	7.96/6.85	1.21/1.10
23	1.65/1.41	1.77/0.95	0.88/1.28	5.26/4.03	0.69/0.52	28	1.99/1.84	1.61/1.54	1.35/1.26	5.84/5.16	0.81/0.68
23	1.55/1.29	1.54/1.12	1.01/0.95	4.85/3.81	0.67/0.51	29	2.13/1.88	1.56/1.44	1.55/1.57	7.44/5.97	1.20/0.94
24	1.31/1.21	1.73/1.05	0.93/1.29	4.26/4.32	0.66/0.60	29	1.95/1.68	1.79/1.56	1.68/1.43	8.56/7.05	1.51/1.21
24	1.87/1.66	1.44/1.34	1.17/1.15	5.55/5.24	0.76/0.69	29	2.11/2.08	1.85/1.33	1.19/1.70	7.04/6.55	0.94/0.86
24	1.82/1.72	1.76/0.89	1.14/1.32	5.63/4.89	0.81/0.71	29	2.25/2.33	1.77/1.50	1.23/1.53	7.55/6.34	1.11/0.86
24	1.61/1.89	1.54/1.15	1.01/1.25	5.29/5.54	0.76/0.61	29	2.13/1.88	1.56/1.34	1.55/1.57	7.44/5.97	1.20/0.94
24	1.47/1.59	1.35/1.30	1.34/1.16	5.42/4.65	0.78/0.64	30	1.95/1.81	1.99/1.41	1.76/1.46	7.51/5.48	1.18/0.81
24	1.65/1.81	1.41/1.44	1.37/1.04	5.58/5.29	0.78/0.72	30	2.20/1.66	1.63/1.80	1.58/1.32	6.24/6.93	1.29/1.24
25	1.37/1.49	1.50/1.39	1.18/1.36	5.26/4.92	0.77/0.69	30	1.82/1.72	1.76/0.89	1.34/1.72	5.63/4.89	0.99/0.71
25	2.03/1.88	1.56/1.04	1.55/1.57	7.44/5.97	1.20/0.94	31	1.96/1.82	2.21/1.43	1.49/1.77	7.89/7.02	1.16/0.97
25	1.82/1.72	1.76/1.19	1.14/1.72	5.63/4.89	0.81/0.71	32	1.88/1.93	2.17/1.68	1.35/1.91	7.78/7.95	1.14/1.24
25	1.49/2.13	1.54/1.37	1.29/1.45	5.95/6.17	0.87/0.89	32	2.30/2.32	2.13/1.78	1.45/1.83	8.98/11.06	1.80/2.15
25	1.72/1.37	1.78/1.28	1.04/1.42	5.31/3.94	0.78/0.55	33	2.10/2.23	1.95/1.87	1.56/2.02	8.37/10.30	1.26/1.42
26	2.05/1.86	1.76/1.63	1.44/1.74	7.86/7.58	1.24/1.27	33	2.38/2.14	1.86/2.07	1.53/1.79	7.25/8.89	1.14/1.93
26	1.63/1.75	1.56/1.52	1.55/1.13	6.04/5.87	0.81/0.83	33	1.94/2.19	2.43/1.92	1.81/2.19	11.62/12.01	1.99/2.21
26	1.74/1.82	1.61/1.44	1.37/1.36	6.18/5.63	0.79/0.75	34	2.20/2.17	2.42/1.95	1.81/2.23	12.39/12.21	2.09/2.24
26	1.82/1.72	1.76/0.89	1.14/1.72	5.63/4.89	0.81/0.71	34	1.99/2.04	1.80/2.05	1.91/1.86	8.90/8.20	1.28/1.24
26	2.11/2.08	1.85/1.33	1.19/1.70	7.04/6.55	0.94/0.86	34	2.27/2.38	1.87/1.73	1.70/1.73	10.28/9.21	1.83/1.58
27	1.89/1.87	2.00/1.38	1.37/1.77	7.92/6.05	1.14/0.86	35	2.60/2.52	2.13/2.08	1.75/1.83	11.98/11.06	2.12/2.15
27	2.13/2.08	1.80/1.45	1.47/1.74	9.32/8.45	1.47/1.25	36	2.46/2.57	2.33/1.74	1.72/2.10	13.08/11.37	2.28/2.10
27	1.71/1.72	1.52/1.65	1.65/1.19	5.23/5.78	0.82/0.84	37	2.41/2.44	2.34/1.81	1.72/2.26	11.49/10.74	1.93/1.94
27	1.68/1.83	1.48/1.34	1.43/1.44	5.14/5.29	0.78/0.72	37	2.53/2.42	2.23/2.00	2.09/1.89	13.34/12.16	2.11/2.27
27	1.71/1.71	1.58/1.28	1.57/1.36	6.65/5.62	1.02/0.87	38	2.55/2.36	2.49/1.91	1.87/2.14	12.41/11.98	2.19/2.40
27	1.90/1.88	1.98/1.65	1.27/1.86	8.50/7.67	1.35/1.05	40	2.76/2.71	2.59/1.97	1.96/2.19	12.48/11.90	2.23/2.11
28	1.97/1.77	2.07/1.37	1.13/1.52	7.42/5.43	1.08/0.77	40	2.61/2.85	2.63/2.21	2.20/2.40	13.65/12.73	2.11/2.27

GA (weeks); AH, AL, AW (cm); AS (cm^2^); AV (cm^3^).

We employed a GLM, with GA as the confounding variable, to analyze the effect of gender on different measurements of the fetal adrenal gland. No statistically significant differences (*p*>0.05) were found in the measurements between the female and male fetuses.

## Discussion

### The Significance of Studying Fetal Adrenal Gland

Since the dimensions of the fetal developing structures, especially in early fetal life, change rapidly, morphometric normative data are well known to play a pivotal role in prenatal US evaluation [15]. Similarly, in prenatal MR imaging, in addition to the morphological subjective evaluation of the structures, quantitative data may be crucial in the diagnosis of anomalies [11]–[15]. Although many reports have been reported about fetal adrenal gland with autopsy [16], in vivo US [4]–[6] and MRI [1], [2], few studies have obtained the normal values of the fetal adrenal gland development with MRI of high magnetic strength.

In this study, the AV of 23–40 weeks GA are about 0.5 cm^3^ smaller than that of Chang’s obtained with 3D US [6]. Our data were obtained with accurate 3D reconstruction models based on manual segmentation from 3.0T MRI of high resolution. It is thought these results may be more accurate than that from 2D or 3D US. Our results may be valuable for the early diagnosis of abnormal adrenal gland development or its tumors on US and in vivo fetal MRI.

### The Superiority of Post-mortem MRI and 3D Visualization of the Fetal Adrenal Gland

This study was carried out on the T_2_-weighted MRI, because we found that the adrenal gland is better depicted on it. The inner part was described with high signal intensities and the outer part was low signal intensities, which was consistent with that of in vivo MRI [1], [2].

It is thought that post-mortem MR imaging is a useful way of measuring the volume of the fetal adrenal gland development and may prove valuable in assessing fetal anomalies in the future. Compared with US, post-mortem MRI is free from the influences of maternal organs, pulse of arteries and movements of the fetus. The growth curve generated in our results can be considered as a model for clinical application.

The fetal upper abdomen was wrapped in the wrist coil in our research, which is more suitable in demonstrating the adrenal gland especially for the small ones. High resolution images can be obtained with a smaller coil and more repetitions of number of excitations (about 30 minutes each for the T_2_ weighted 3D SPGR sequence), so that our results may be convincing.

The 3D reconstruction analysis has several advantages over traditional pathologic analysis. First, it provides images of the fetal adrenal gland surface and inner structures without having to excise it, thus precluding deformity during excision and produced by gravity. Second, arbitrary slices can be obtained by post-processing. Third, accurate measurements of each component can be easily and exactly obtained with the organs in situ [17]. However, the results will be definitely influenced when they are removed out from the abdomen. Post-mortem MRIs of high quality are sufficient for segmentation, reconstruction and later quantitative analysis. These three dimensional visualization models can exactly delineate the developmental changes of fetal adrenal gland during the second half of gestation, and may supply great help in clinics.

### Asymmetry and Sexual Dimorphism of the Fetal Adrenal Gland

Nowak D et al [16] have discovered that there were asymmetries (slightly higher for the left than the right) and sexual dimorphisms (slightly higher for females than for males) in the parameters of the fetal adrenal glands between the 4th and 7th months of gestation. In our result, we also demonstrated that the AW, AH, AS and AV of the left fetal adrenal gland were larger than that of the right one. However, no statistically significant differences (*p*>0.05) were found in the measurements between the female and male fetuses in our research.

The inconsistencies may be explained as follows. First, the definition of fetal age (in weeks or in months) is different. The fetal age in this study is expressed by weeks, which is most commonly used now, but not by months as that of Nowak D’s [16]. Second, the samples size in this study is relatively small, and the GA span of the male and female specimens and their distributions in each week is uneven. The above reasons will definitely affect the analysis of asymmetries and sexual dimorphisms. It can only be suspected that there are asymmetries but no sexual dimorphisms of the fetal adrenal gland in the second half of pregnancy.

### Limitations

There are some limitations for this study, first, the selected specimens did not have detected fetal adrenal gland abnormalities, but there was a theoretical possibility that an undetected anomaly may exist. It is thought that our inclusion criteria may reduce number of these fetuses to the least. Second, relative to the sample size in other US and in vivo fetal MRI studies, the sample size of 52 fetuses in this study was small, and our sample suffered from a lack of data points at 30–40 weeks GA. It is thought additional measurements would have been statistically unlikely to alter the shape of the defined lines because of the close fitting of the model lines of our data. Third, the scanned fetuses had undergone formalin fixation, which would slightly affect the measurements, but previous studies [10]–[14], [17] have ascertained the clinical application of measurements obtained on the basis of post-mortem brain tissues.

## Conclusions

Our data precisely delineate the normal trajectory of fetal adrenal gland development in the second half of gestation, and may be valuable in clinical settings.

## Supporting Information

Movie S1 **The 3D visualization model of the fetal adrenal gland of 32 weeks GA.**
(AVI)
